# Preformulation Studies on Piperlongumine

**DOI:** 10.1371/journal.pone.0151707

**Published:** 2016-03-16

**Authors:** Alhassan Aodah, Aaron Pavlik, Kelly Karlage, Paul B. Myrdal

**Affiliations:** Department of Pharmacy Practice and Science, College of Pharmacy, University of Arizona, Tucson, Arizona, United States of America; Tokushima Bunri University, JAPAN

## Abstract

Piperlongumine is a natural alkaloid extracted from piper plants which has been used traditionally for the treatment of certain diseases. This compound shows interesting *in vitro* pharmacological activity such as selective anticancer activity and higher cytotoxicity than methotrexate, cyclophosphamide and adriamycin on breast, colon, and osteosarcoma cancers, respectively. However, the physicochemical properties for this compound have not been well characterized. In this research, preformulation studies for piperlongumine have been performed to determine factors which influence solubility and stability which, in turn, can be used to assist future formulation development. The solubility of piperlongumine in water was found to be approximately 26 μg/ml. Using 10% polysorbate 80 as a surfactant resulted in a 27 fold increase in solubility. Cosolvents and cyclodextrins afforded concentrations of 1 mg/ml and higher. The pH degradation rate profile for piperlongumine at various temperatures shows significant instability of the drug at pH values ≥ 7 and 3, and maximum stability around pH 4. It was estimated that it would take approximately 17 weeks for piperlongumine to degrade by 10% at 25°C, pH 4. Additionally, piperlongumine showed marked photo-degradation upon exposure to an ultraviolet light source, especially in aqueous media.

## Introduction

**Fig 1:**
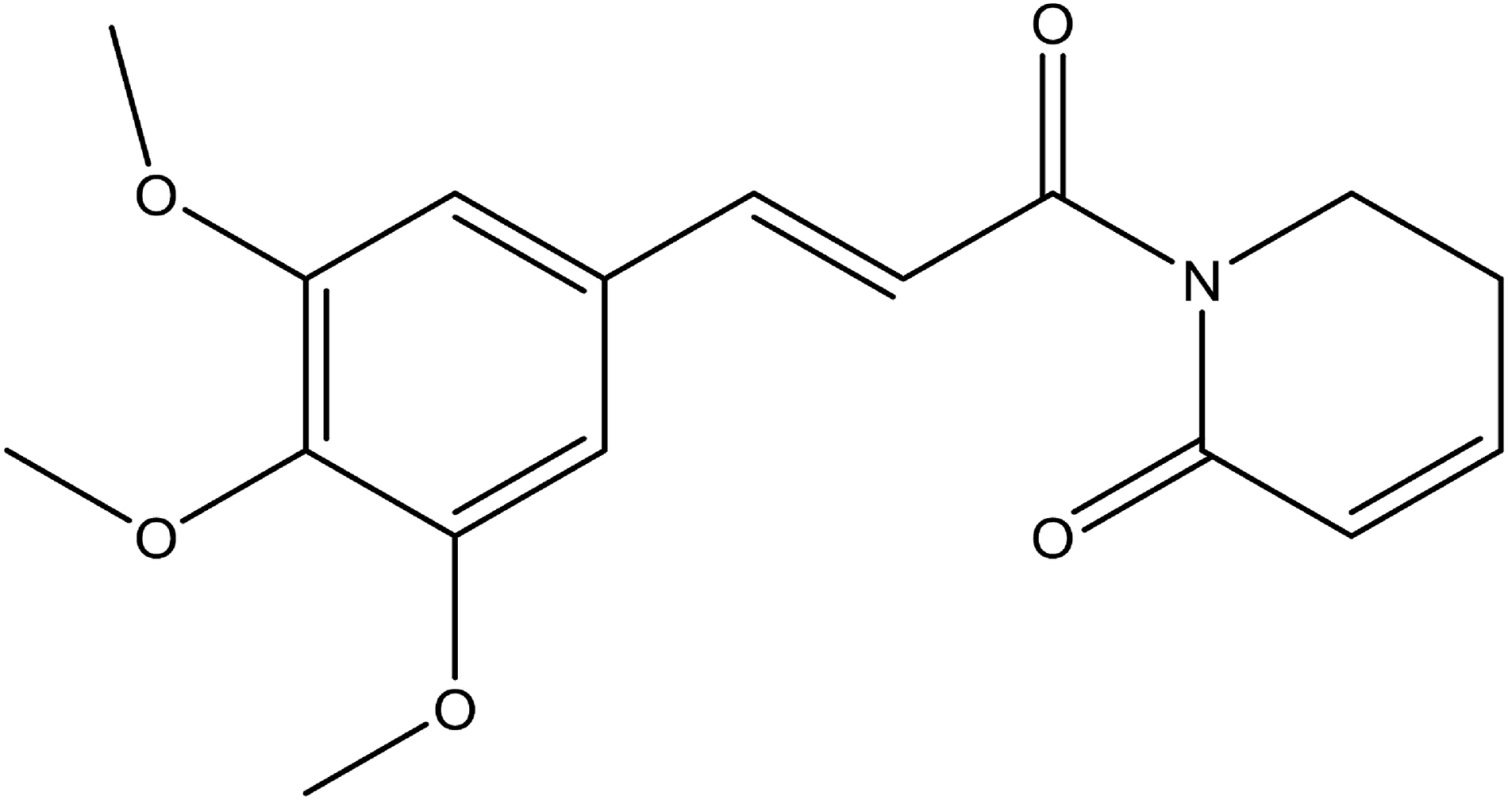
The chemical structure for piperlongumine.

Piperlongumine, or piplartine, 1-[(2E)-3-(3, 4, 5-trimethoxyphenyl) prop-2-enoyl]-1, 2, 5, 6-tetrahydropyridin-2-one, (Fig 1), is a historically interesting natural alkaloid compound as piper plants have been used in traditional Ayurvedic system medicine to treat some tumors and other diseases such as malaria, gonorrhea, bronchitis, asthma, and cough [1]. First isolated from the piper plant in 1961, the chemical structure was elucidated in 1963 [2].

Piperlongumine has become a compound of interest in recent years as it exhibits an array of promising anticancer activities. This compound shows selective cytotoxicity toward cancer cells [3], as well as demonstrating higher cytotoxic activity on breast, colon, and osteosarcoma cancer cells of patient derived cell line growth in comparison with other anticancer drugs like methotrexate, cyclophosphamide and adriamycin [4]. While the molecular mechanisms behind piperlongumine’s cytotoxicity are still being elucidated, it has been shown to induce cell death by enhancing reactive oxygen species and DNA damage for several types of cancer i.e., head and neck [5], pancreatic [6], and melanoma [7], as well as inhibiting CRM1, a major nuclear exporter protein [8]. Additionally, piperlongumine has been used to inhibit signal transducer and activation of transcription 3 (STAT3) in order to induce anoikis in anoikis resistant melanoma and pancreatic cells lines [9, 10]. As published in Nature in 2011, piperlongumine also manifests antiangiogenic effects via its ability to reduce vascular endothelial growth factor (VEGF) and CD31 kinases in cancer cells, which in turn inhibits the formation of new blood vesicles for tumor cells, as well as its ability to work as an antimetastatic agent for the cancer cells [11]. As well as being a successful single agent, several studies have explored the use of piperlongumine in combination with other agents such as docetaxel [12], cisplatin [5], and tumor necrosis factor-related apoptosis-inducing ligand (TRAIL) [13], further demonstrating the utility of this compound as an anticancer agent.

In addition to promising anticancer activities, other pharmacological activities include inhibition of the formation of artherosclerosis plaque in vivo, [14] and in vitro antibacterial activity on Pseudomonas aeruginosa, Klebsiella pneumonia, and Staphylococcus aureus [15]. A recent review by Bezerra et al. highlights many of these activities as well as piperlongumine’s ability to act as an inhibitor of platelet aggregation, anxiolytic and antidepressant, and antidiabetic [16]. These activities, especially anticancer, makes piperlongumine an appealing compound for further development. However, to this date, very few studies have focused on the preformulation and/or formulation of this drug. In 2016, Fofaria et al. published their findings for the preparation of nanoemulsion formulations of piperlongumine [17]. While this study provides some solubility data for piperlongumine, more preformulation work is warranted.

In this current research, preformulation studies have been conducted to determine the solubility of piperlongumine in water and other systems including cosolvents, surfactants and complexing agents, which are commonly used in various pharmaceutical formulations. Moreover, the aqueous stability of piperlongumine as a function of pH, temperature and ionic strength has been extensively studied for more than 18 months. Photo-stability studies and solid-state characterization were also performed. The results of these essential preformulation studies will aid in the future formulation development for this drug.

## Materials and Methods

### Materials

Piperlongumine was supplied by Research Products International (Mt. Prospect, IL, USA). HPLC grade acetonitrile (ACN) was obtained from Spectrum Chemical Manufacturing (Gardena, CA, USA). USP grade ethanol was obtained from Decon Laboratories Inc. (King of Prussia, PA US). Cremophor Rh 40 was obtained from BASF Aktiengesellschaft, (Ludwigshafen, Germany). Sulfobutyl ether β-cyclodextrin sodium salt research grade (Captisol^®^) was obtained from CyDex Inc. (Lenexa, Kansas, USA). Hydroxypropyl-β-cyclodextrin (Cavasol^®^) was obtained from Wacker Chemie AG (Burghausen, Germany). Polyethylene glycol 400, propylene glycol, glycerin, polysorbate 80 (Tween 80), n-octanol, ascorbic acid, ethylenediaminetetraacetic acid (EDTA) disodium salt, sodium bisulfite, monopotassium phosphate, disodium phosphate, disodium citrate and boric acid were obtained from Sigma Aldrich (St. Louis, MO, USA). Phosphate buffered saline was obtained from Amresco, Inc. (Solon, OH, USA).

### Methods

#### HPLC analysis

The HPLC system consisted of a Waters 2690 separation module (Waters, Milford, MA, USA) coupled with a Waters 996 Photodiode Array (PDA) detector. Analysis was performed by a reverse phase HPLC assay, using a 150 mm × 2.1 mm, Alltima C18 5 μm column (Grace Davison Discovery Sciences, Deerfield, IL), maintained at 30 ± 2°C. Ultraviolet detection was done at 328 nm. Mobile phase conditions were 40:60 (v/v) ACN: H_2_O at a flow rate of 0.3 ml/min. The injection volume was 5 μl. The retention time for piperlongumine was ~6.9 min. Quantification was determined using peak area and calculated from a five-point standard curve. Standards were prepared by volumetric dilution in acetonitrile and stored at 4°C, protected from light.

#### Solubility studies

Solubility of piperlongumine was determined in purified water and the following cosolvent and surfactant systems: 5, 15, 30% (v/v) acetonitrile; 5, 15, 30, 50, 100% (v/v) of polyethylene glycol 400 (PEG 400), ethanol, glycerin and propylene glycol; 3, 7, 10% (w/v) of Tween 80; 2.5, 5, 10% (w/v) of Cremophor Rh 40; and a solution composition of 10% ethanol: 40% PEG 400 (w/v). Additionally, the complexing agents, sulfobutyl ether β-cyclodextrin sodium salt and hydroxyl-propyl-β-cyclodextrin, were examined at concentrations of 5, 10, 20, 40% (w/v).

An excess quantity of raw drug crystals were placed in a known volume of each medium in closed, light protected glass vials and allowed to agitate for at least 24 hours at ambient temperature. Samples were visually inspected to ensure that solid drug was still in excess and then filtered through a 0.2 μm PTFE filter. The filtrate was assayed via the previously described HPLC method and the concentration of piperlongumine in solution was determined.

#### Partitioning studies

Piperlongumine crystals were placed in a small glass bottle containing equal volumes of n-octanol and water. The bottle was shaken for 24 hours, after which time, the bottle was allowed to equilibrate without mixing for an additional 24 hours to ensure full separation between the n-octanol and water layers. A sample from each layer was carefully withdrawn and analyzed by HPLC.

#### Solid state characterizations

Thermal analysis was performed with a Q1000 series differential scanning calorimeter (DSC) (TA Instruments, New Castle, DE, USA). Indium was used for the calibration of the DSC. Samples of 4 to 5 mg were weighed out and placed in an aluminum pan and crimped with an aluminum lid. Samples were equilibrated and isothermally heated at 30°C for 5 min, followed by heating at 2°C/min up to 150°C. A nitrogen purge was used at 40 ml/min for each sample.

Thermogravimetric analysis (TGA) was performed on a TA Instruments Q50 TGA. Samples weighing 4 to 5 mg were placed in an empty aluminum pan, covered with an aluminum lid, crimped and pin perforated. The pan was then heated at 5°C/min up to 150°C. Weight loss as a function of temperature was analyzed under a nitrogen purge at 60 ml/minute.

Powder X-ray diffraction patterns were obtained with a Philips PANalytical X’pert Pro MPD instrument equipped with an X’Celerator detector. Cu Kα radiation was used (λ = 1.5418 Å). Powdered samples were smoothed onto a zero background silicon wafer and rotated at 1 revolution/8 seconds during data collection. The scan time was 1.5 hours.

#### Aqueous stability studies

Aqueous stability studies for piperlongumine were conducted in three buffer systems: citrate (pH 3, 4, and 5); phosphate (pH 5, 6, and 7); and borate (pH 8 and 9). The ionic strength for the solutions was also varied, utilizing approximately 0.2 or 0.5 M. Each buffered sample was prepared with 10% acetonitrile as a cosolvent, then sealed and protected from light. Samples were stored at four different temperatures: 4°C (for samples at pH 3, 8 and 9 only), 26°C, 56°C and 67°C. Aliquots were regularly withdrawn for analysis. Changes in pH, if any, were also monitored. Stability studies were carried out for 18 months or until complete degradation occurred.

#### Photo-stability

Photo-stability was examined by preparing four groups of samples, each containing a triplicate sample and one reference (protected from light). These groups covered high and low drug concentrations in the following compositions:

- 150 μg/ml of drug in 100% acetonitrile
- 150 μg/ml of drug in 15:85 acetonitrile: water
- 30 μg/ml of drug in 100% acetonitrile
- 30 μg/ml of drug in 15:85 acetonitrile: water

Each sample was prepared in a transparent glass vial and closed without any entrapped air. A reference sample was utilized for each group by covering one of the vials with aluminum foil to protect it from the light exposure. All vials were exposed for 165 min using Sun 340 sunlamps yielding UV emission between 295 and 390 nm to include UVA and UVB wavelengths. Samples were placed in an area where the UV intensity was measured to be 1.57 mw/cm^2^. After the exposure time, samples were analyzed using the HPLC method described previously.

#### Additional stability studies

The addition of antioxidants such as ascorbic acid and sodium bisulfite and the chelator EDTA were evaluated with the drug at pH 3 (citrate buffer) and pH 7 (phosphate buffer). Samples in phosphate buffer were maintained at 26°C while the samples in citrate buffer were kept at 56°C. All three agents were used at a concentration of 0.05% w/v. Aliquots were withdrawn on a regular basis and pH and drug concentration were monitored. Piperlongumine stability was also examined after the addition of 1.5% w/v hydrogen peroxide in pH 3 citrate buffer at room temperature.

#### LC-MS analysis

Stability samples which showed degradation, as evidenced by new peaks appearing in the HPLC chromatograms, were submitted for LC-MS analysis, in order to identify the masses of the degradation products. Samples were separated on an Altima HP C18, 5 μm, 150 x 2.1 mm column (Grace Davison Discovery Sciences, Deerfield, IL) using a Paradigm MS4B—multi-dimensional separations module (Michrom BioResources, Inc., Auburn, CA). The mobile phase (40% ACN:H_2_O) was delivered isocratically at a flow rate of 300 μl/min. A 20 μl injection loop was used to introduce 2 μl of sample diluted with 18 μl of the mobile phase. The AB SCIEX API 3000 triple-quadrupole mass spectrometer (Applied Biosystems, Foster City, CA) was controlled by Analyst 1.5.1 and used in-line with the HPLC. Mass spectrometric analysis was performed using both MS (full scan) and MS/MS (product ion) type in positive mode with an APCI source. Instrument-specific parameters were as follows: source temperature, 350°C; declustering potential (DP), 20 V; entrance potential (EP), 10 V; collision energy (CE), 10 V.

## Results and Discussion

### Chromatography

**Fig 2:**
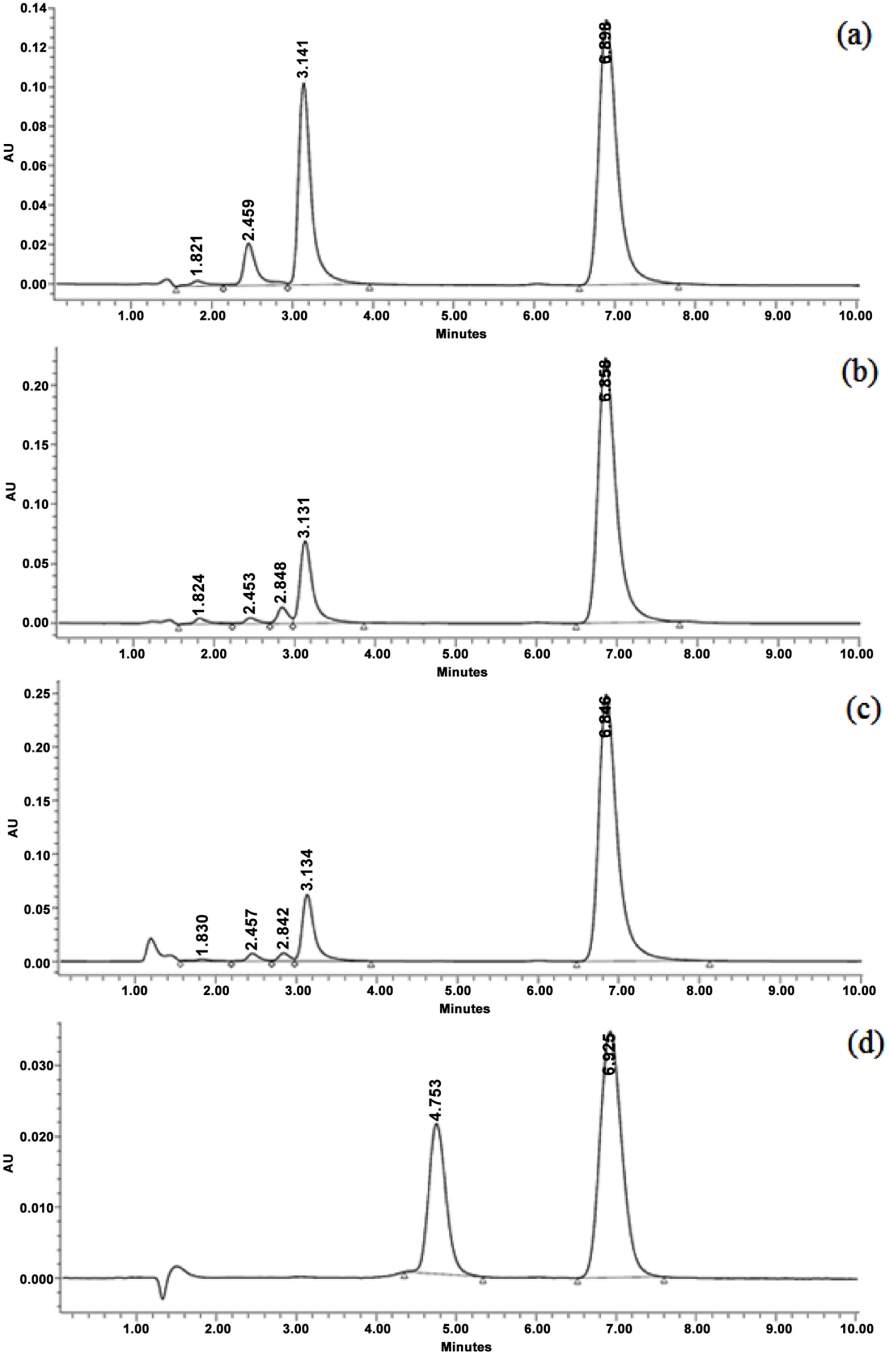
HPLC chromatograms for the separation of piperlongumine (peak at 6.9 minutes) in different stability conditions. (a) Sample at pH 8. (b) Sample at pH 5. (c) Sample at pH 3. (d) Photo-stability sample.

The isocratic HPLC method developed eluted piperlongumine within a 10 minute run time, giving a Gaussian shaped peak for the drug at ∼ 6.9 minutes. Furthermore, utilizing this method, piperlongumine was completely resolved from the degradation products which resulted from the stability studies as seen in Fig 2. The method was shown to be linear over the working concentration range of 0.5 to 120 μg/ml. The same HPLC method was used to analyze all samples from solubility, thermal and photo-stability studies.

### Solubility Studies

**Fig 3:**
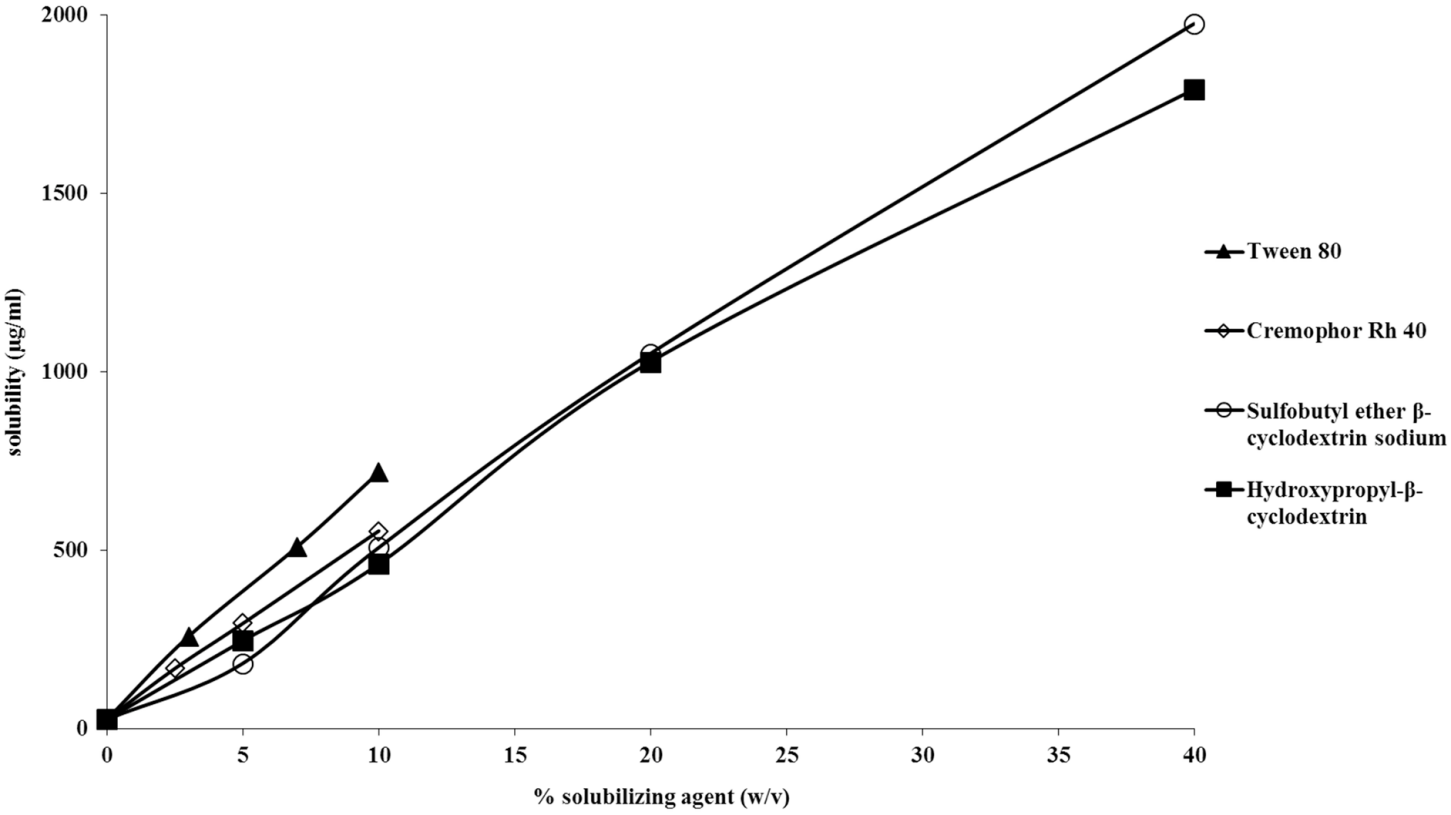
Solubility of piperlongumine in different surfactant and complexing systems.

**Fig 4:**
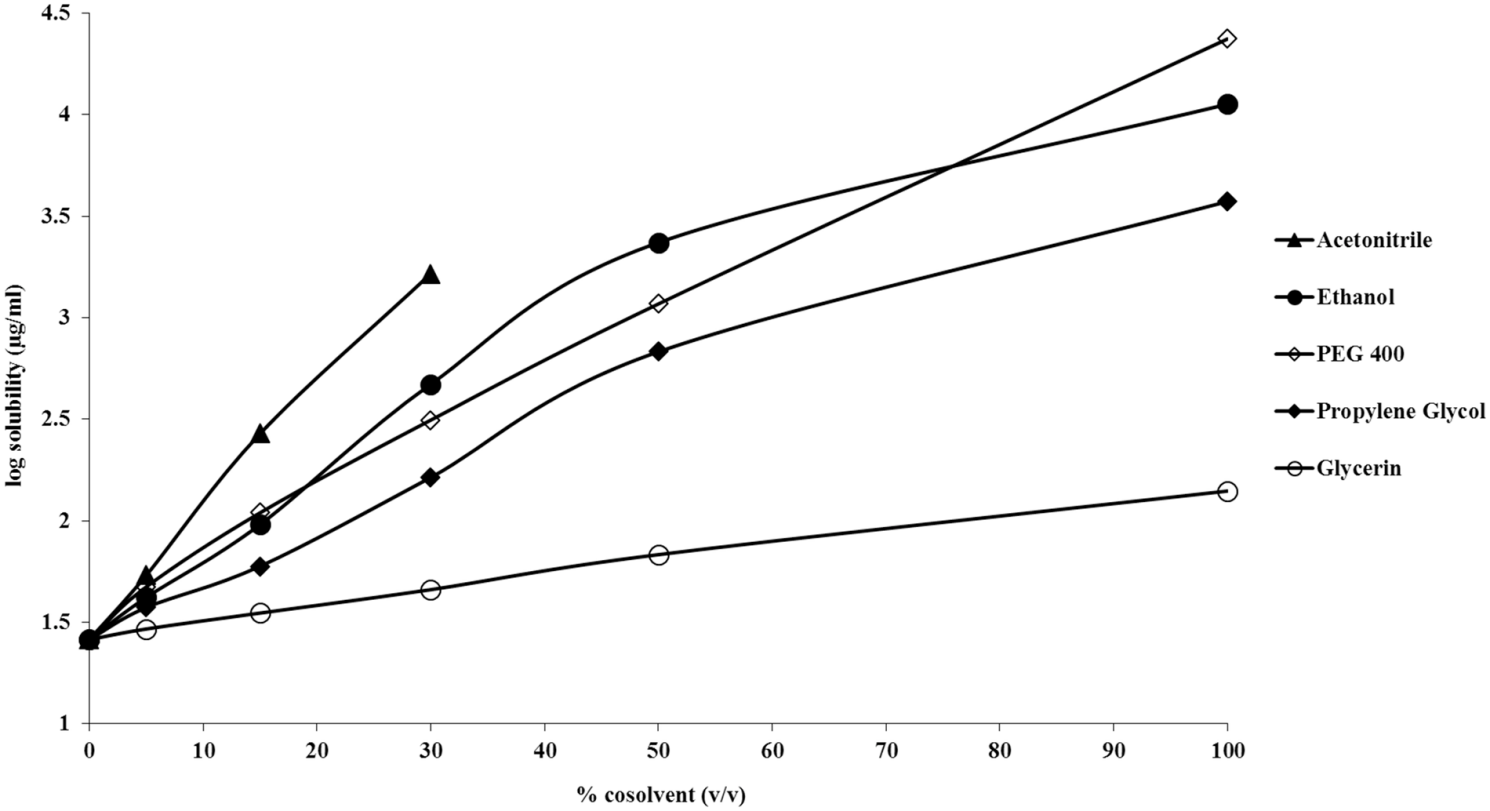
Solubility of piperlongumine in different cosolvent systems.

Experimental measurement of the aqueous solubility of piperlongumine indicated that the drug has an intrinsic solubility of 26 ± 2.9 μg/ml. This experimental solubility value is about ten times less than that determined by Fofaria et al [17]. Water solubility does not change as a function of pH. Solubilization studies were also performed utilizing surfactants, cyclodextrins and cosolvents. The results are shown in Figs 3 and 4. The cyclodextrin and surfactant systems demonstrate linear solubilization profiles (Fig 3), while the cosolvent systems followed predominantly log-linear profiles (Fig 4). It was found that 10% Tween 80 was capable of enhancing piperlongumine solubility up to 27 fold (solubility of approximately 700 μg/ml). A 10% (w/v) Cremophore Rh 40 system solubilized approximately 550 μg/ml. Hydroxypropyl-β-cyclodextrin and sulfobutyl ether β-cyclodextrin both solubilized about 1 mg/ml at a 20% (w/v) concentration. While acetonitrile was the most efficient cosolvent, with 30% improving the drug solubility by 63 fold (1.6 mg/ml), its utility is limited to analytical uses. Of the cosolvents utilized, ethanol and PEG 400 were the best cosolvents for piperlongumine. At lower concentrations the solubilization was similar for both ethanol and PEG 400. At 50% cosolvent, ethanol solubilizes nearly twice as much as PEG 400 (~ 2.3 mg/ml and ~1.1 mg/ml, respectively). Interestingly, in pure solvent, piperlongumine has better solubility in PEG 400 than in ethanol (~22 mg/ml and ~11 mg/ml, respectively). Fofaria et al. [17] also observed that piperlongumine is more soluble in PEG 400 as compared to ethanol. By utilizing a 10% ethanol: 40% PEG 400 (w/v) composition, a solubility of approximately 1.7 mg/ml was obtained.

Three piperlongumine prototype formulations were evaluated for precipitation upon dilution. These formulations were a 500 μg/ml in a 10% (w/v) Tween 80 formulation, a 1 mg/ml in a 10% ethanol: 40% PEG 400 (w/v) formulation, and a 1 mg/ml in a 20% (w/v) hydroxypropyl-β-cyclodextrin formulation. All three were serially diluted from a 1:1 to a 1:10 by adding either 1 ml of water or 1 ml of phosphate buffered saline (PBS) to the formulation. After the addition of each ml of either water or PBS, the mixture was evaluated for precipitation. This was repeated for each sequential addition. No precipitation was observed for any of the dilutions for any of the three formulations. However, a small amount of precipitation was observed after 24 hours for dilutions with the 10% ethanol: 40% PEG 400 (w/v) formulation.

### Partitioning study

Piperlongumine exhibits lipophilic properties when evaluated in an n-octanol/water partition coefficient study. The partition coefficient (log P) was experimentally determined to be 2.37 ± 0.12 at room temperature.

### Solid-state characterization

**Fig 5:**
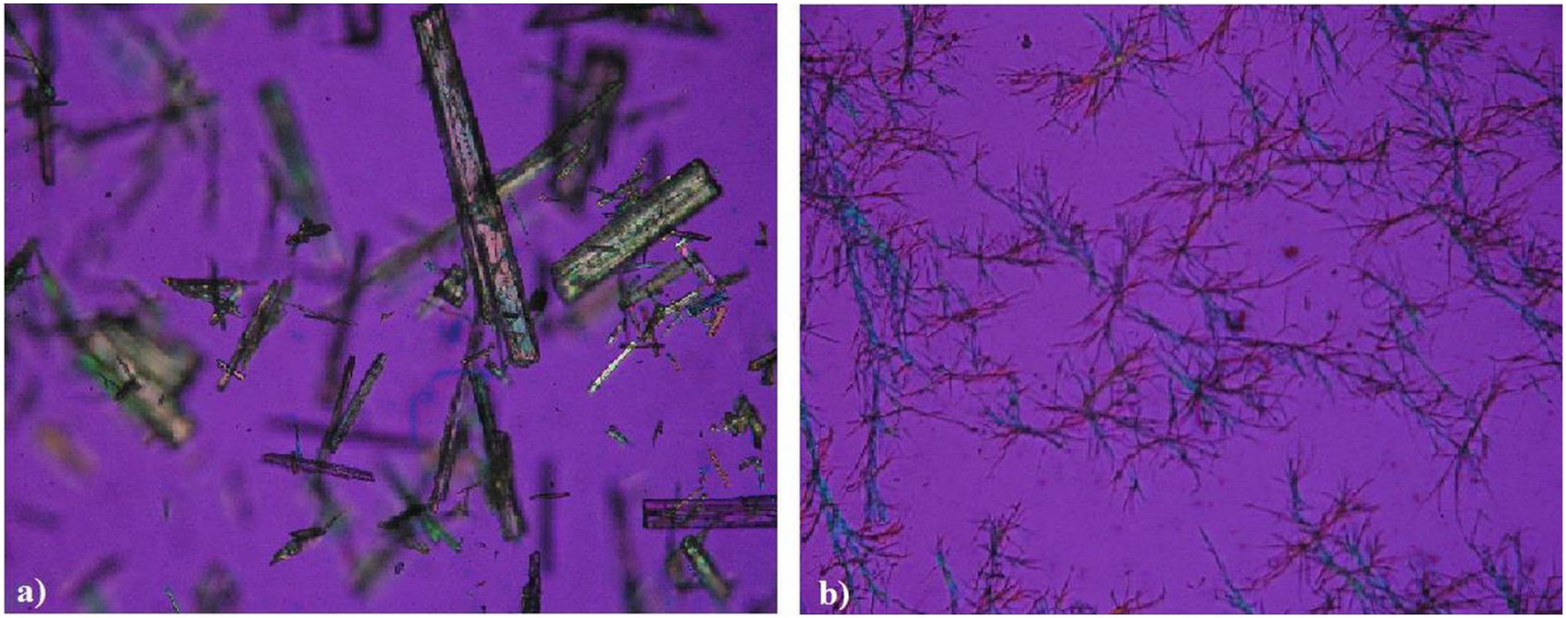
Photomicrographs of piperlongumine (400X). (a) Original crystals, (b) Crystalline precipitant.

It was observed upon microscopic evaluation that piperlongumine crystals were shaped like rectangles (Fig 5a). Using hot stage microscopy, these crystals start melting at 123°C, and are completely melted at 126°C. Examination of different re-crystallized forms of piperlongumine such as: precipitate of the drug in water, ethanol, methanol and acetonitrile, demonstrated that all conditions give the same irregular needle like crystal morphology (Fig 5b).

**Fig 6:**
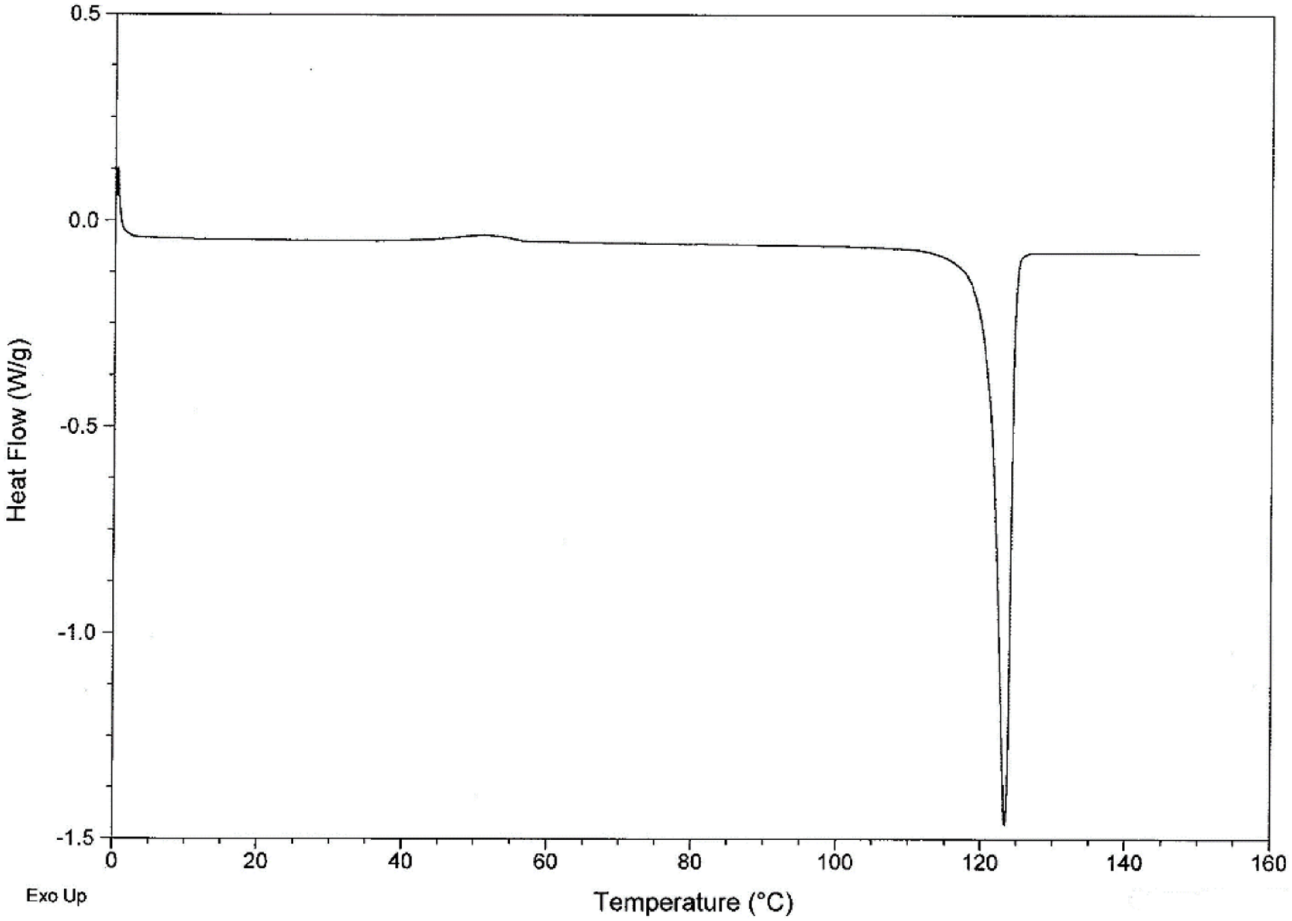
DSC thermogram of piperlongumine.

Analysis of piperlongumine crystals by DSC gave an endothermic peak with an onset of 109.6°C and a maximum of 123.3°C (Fig 6). TGA analysis for piperlongumine crystals showed no weight loss occurred until the melting point was reached.

Piperlongumine precipitated from supersaturated solutions in water, ethanol, methanol and acetonitrile was examined by DSC. Although the recrystallized crystals had different crystal habits, they had the same melting point as the original drug crystals.

**Fig 7:**
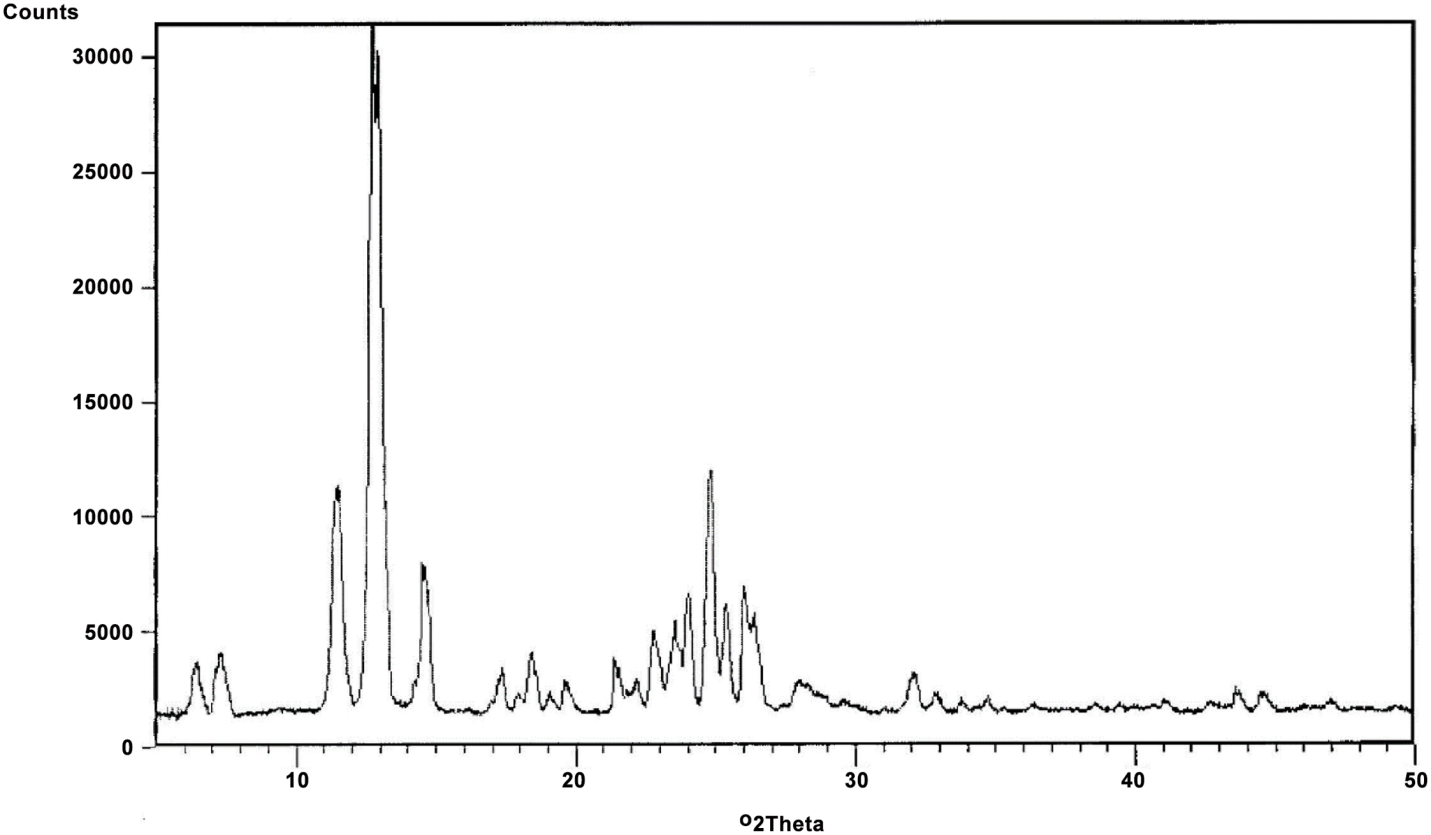
Pattern of X-ray diffraction of piperlongumine crystals.

Analysis of crystals utilizing X- ray powder diffraction supports that piperlongumine exists as a specific crystalline solid (Fig 7). The X-RPD chart shows long range molecular order. This result is in accordance with the results of the calculated single crystal data published by Banerjee and Chaudhuri in 1985 [18].

### Stability Studies

**Fig 8:**
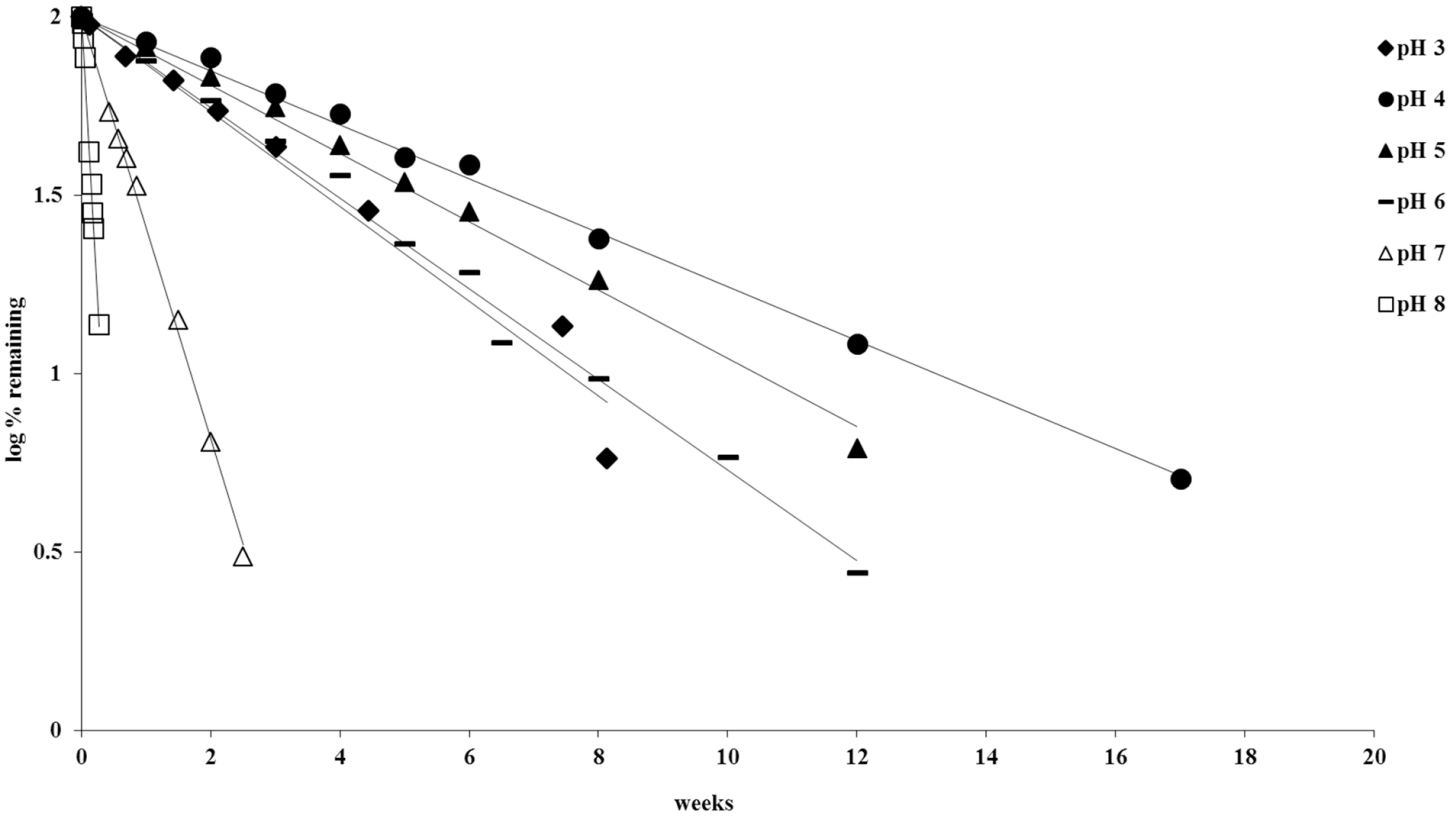
Degradation of piperlongumine as a function of pH at 56°C.

Fig 8 plots the logarithmic percentage of drug remaining verses time at 56°C and shows first order degradation kinetics of thermal degradation of piperlongumine for several pH conditions. Apparent first order degradation was also obtained for the 4, 25 and 67°C degradation profiles. Degradation rate constants, k-values, for piperlongumine were calculated using the slopes of the trend lines of each profile.

**Fig 9:**
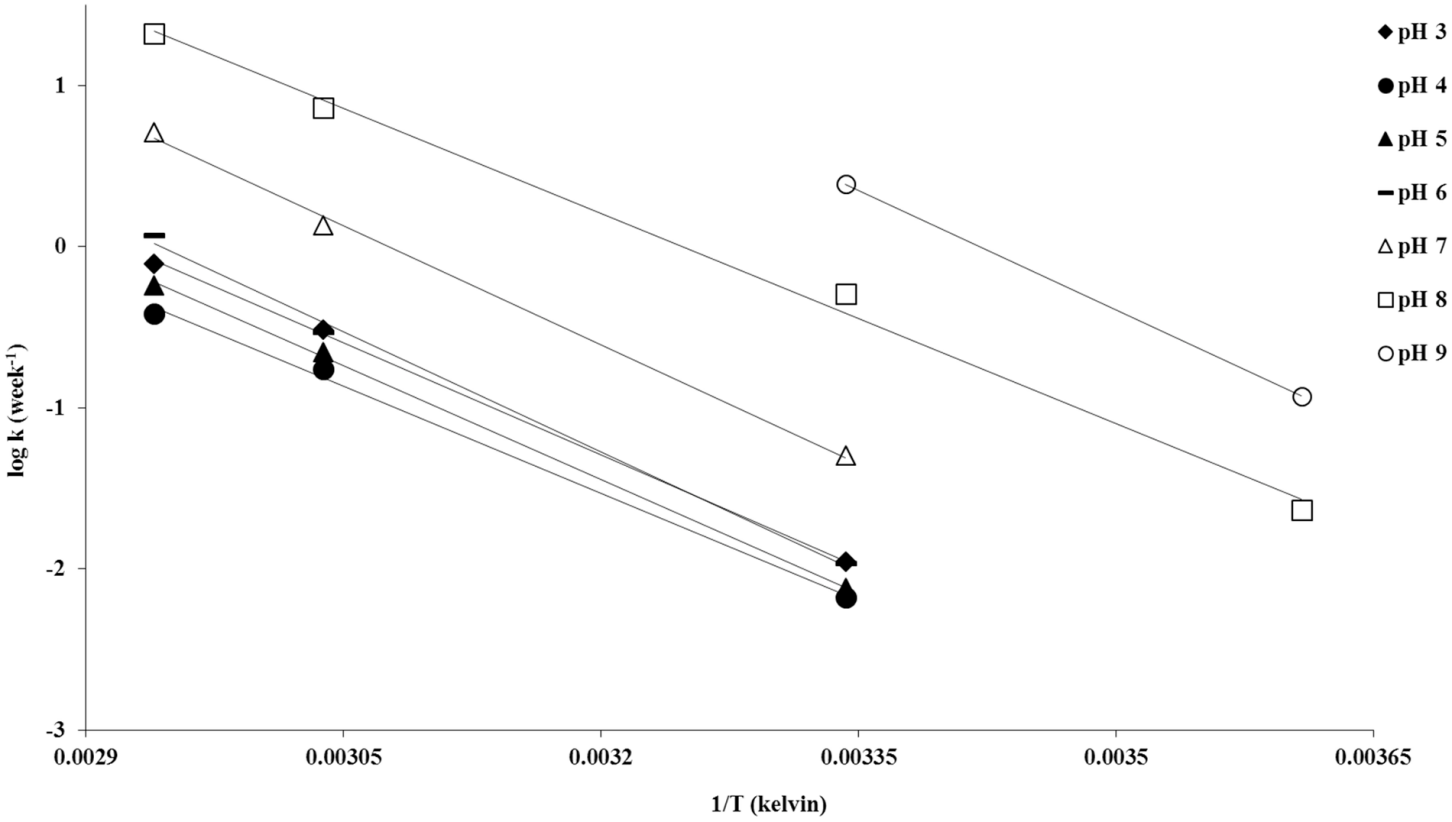
Arrhenius plots for piperlongumine degradation at different pH values.

An Arrhenius plot of log k versus the reciprocal of the temperature (Fig 9) was utilized to determine the length of time it takes for piperlongumine to degrade 50% (T_50_) and 10% (T_90_). For a given pH the Arrhenius equation was utilized:

Log k = log A − (Ea/2.303 RT) (1)

Where:

A is the Arrhenius factor.
Ea is the activation energy (J/mole).
R is the gas constant = 8.314 J/mol. K.
T is the temperature in Kelvin.

Arrhenius activation energies at different pHs were calculated from the slope of each linear curve and by using the Arrhenius equation. For all pHs, the activation energies were within the range of 88–95 kJ/mol. All calculated values are presented in Table 1.

**Table 1:** Summary of stability results.

pH	Buffer type	Ionic strength	k values (weeks^-1^)				Ea (kJ/mol)	T_50_ at 25°C (weeks)	T_90_ at 25°C (weeks)
			4°C	26°C	56°C	67°C			
**3**	citrate	0.2	*	0.0110	0.3057	0.7808	88.5	69.6	10.6
**3**	citrate	0.5	*	0.0101	0.3413	0.8383	92.3	75.4	11.5
**4**	citrate	0.2	---	0.0067	0.1739	0.3813	84.8	112.9	17.2
**5**	phosphate	0.2	---	0.0076	0.2201	0.5741	89.9	101.5	15.4
**5**	phosphate	0.5	---	0.0090	0.1764	0.5741	84.8	88.7	13.5
**5**	citrate	0.2	---	0.0090	0.2045	0.6125	86.7	87.6	13.3
**6**	phosphate	0.2	---	0.0108	0.2919	1.1652	95.0	75.5	11.5
**7**	phosphate	0.2	---	0.0506	1.3607	5.1370	94.0	16.0	2.4
**8**	borate	0.2	0.0232	0.5120	7.2924	20.933	83.0	2.0	0.3
**9**	borate	0.2	0.1182	2.4375	---	---	94.8	0.3	0.05
**9**	borate	0.5	0.1171	2.4372	---	---	95.1	0.3	0.05

*Not determined. Rates were not significantly different than zero over the course of the study.

**Fig 10:**
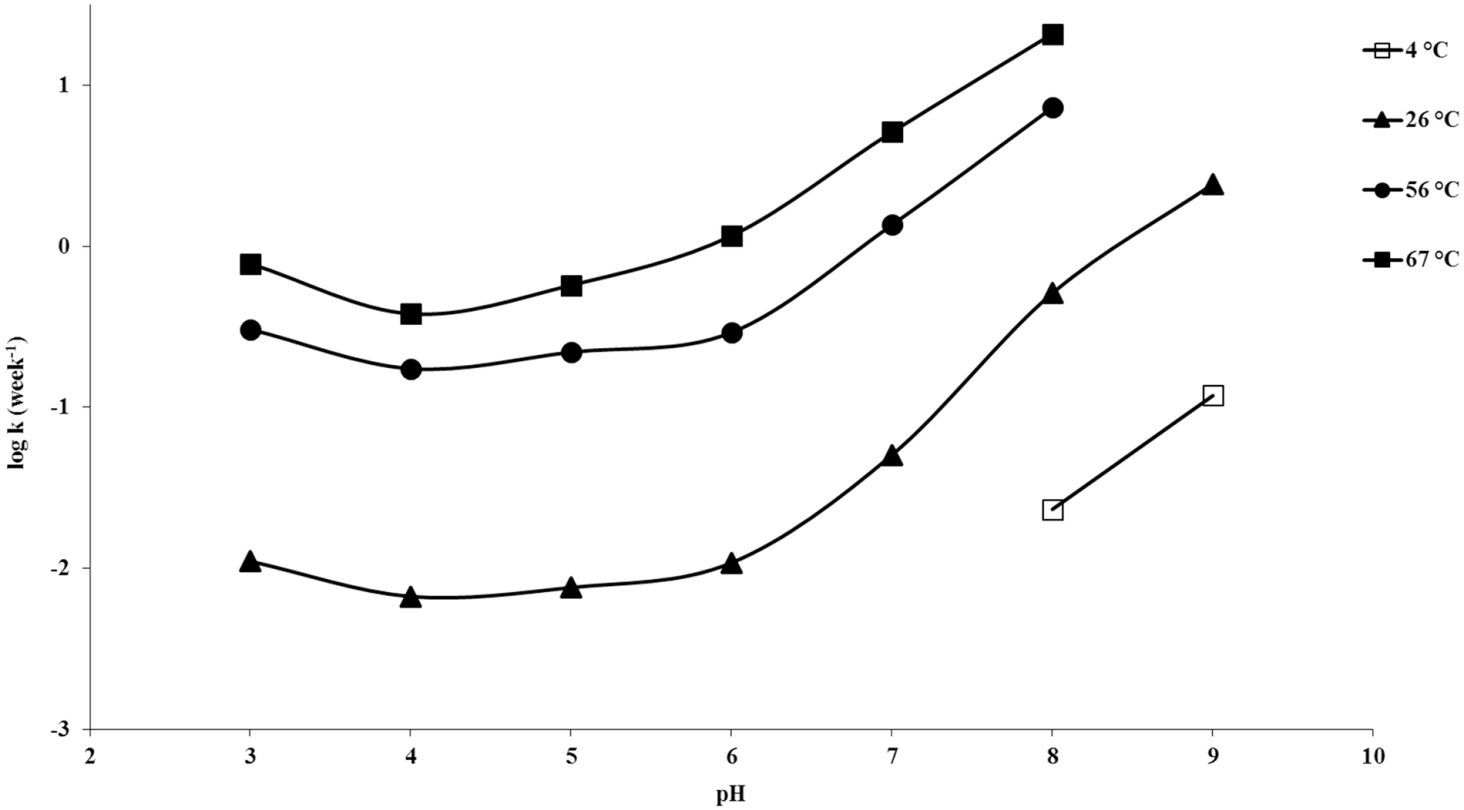
Piperlongumine pH degradation rate profiles. All samples were at an ionic strength of ~0.2 M. Buffer types are: citrate for pH 3 and 4, phosphate for pH 5, 6 and 7, borate for pH 8 and 9.

Fig 10 shows the overall degradation rate vs. pH profile of piperlongumine at 26°C, 56°C and 67°C. From the stability profiles it can be observed that piperlongumine has maximum stability around pH 4; the T_90_ at this pH is about 17 weeks at 25°C

**Fig 11:**
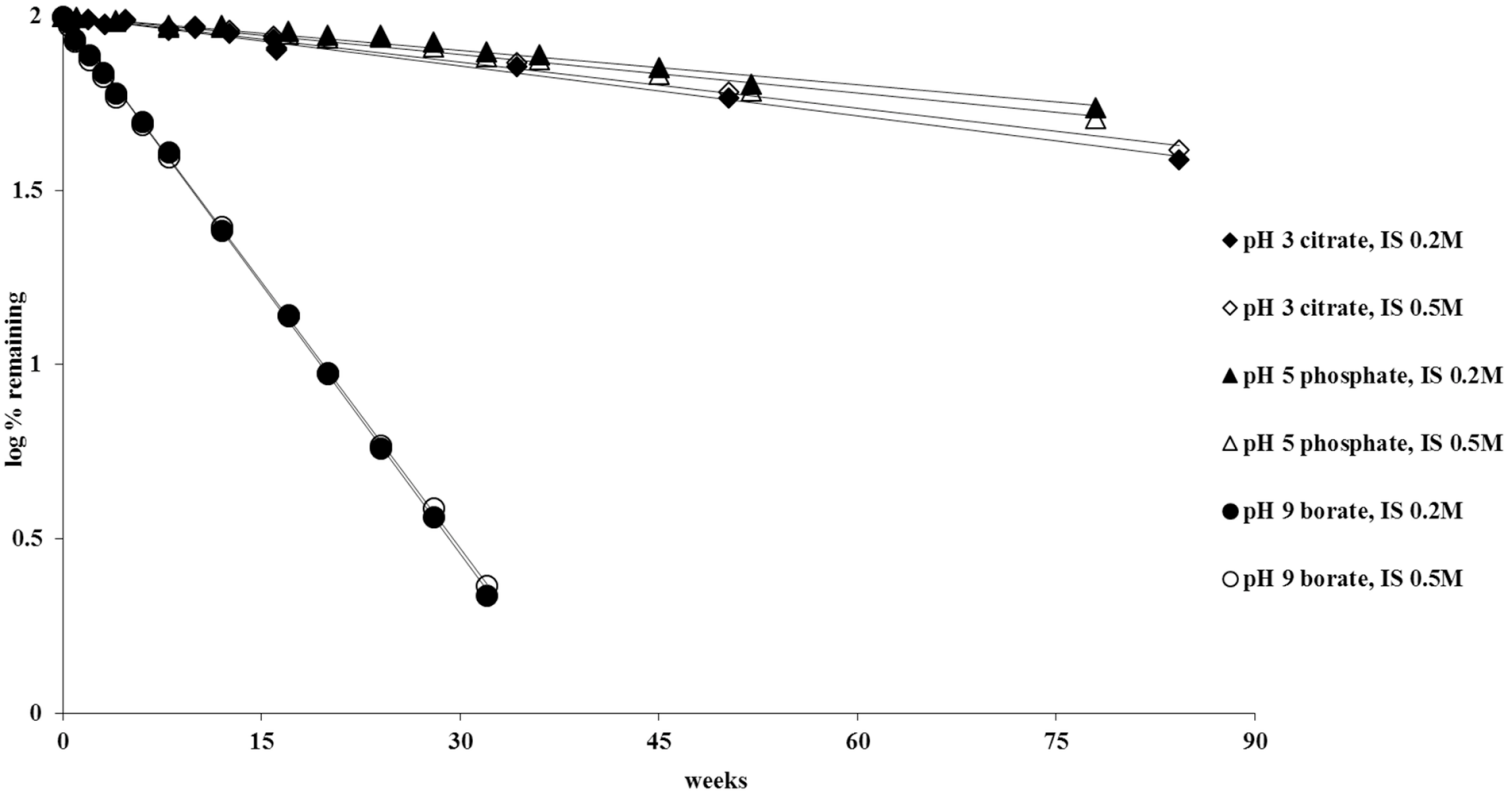
Effect of ionic strength (IS) on stability of piperlongumine at pH 3, 5 and 9 at 26°C.

The effects of ionic strength and buffer species were also studied. As can be seen from the data presented in Table 1 and shown in Fig 11, an increase in ionic strength from approximately 0.2 M to 0.5 M had nominal effects (and likely not significant) on the stability half-life of piperlongumine at pH 3 and pH 5. No difference in degradation rate was observed between the 0.2 M to 0.5 M ionic strength samples at pH 9 in a borate buffer. Two different buffers, citrate and phosphate, were evaluated at pH 5. As can be seen in Table 1, the degradation rates are similar for both buffer systems.

**Fig 12:**
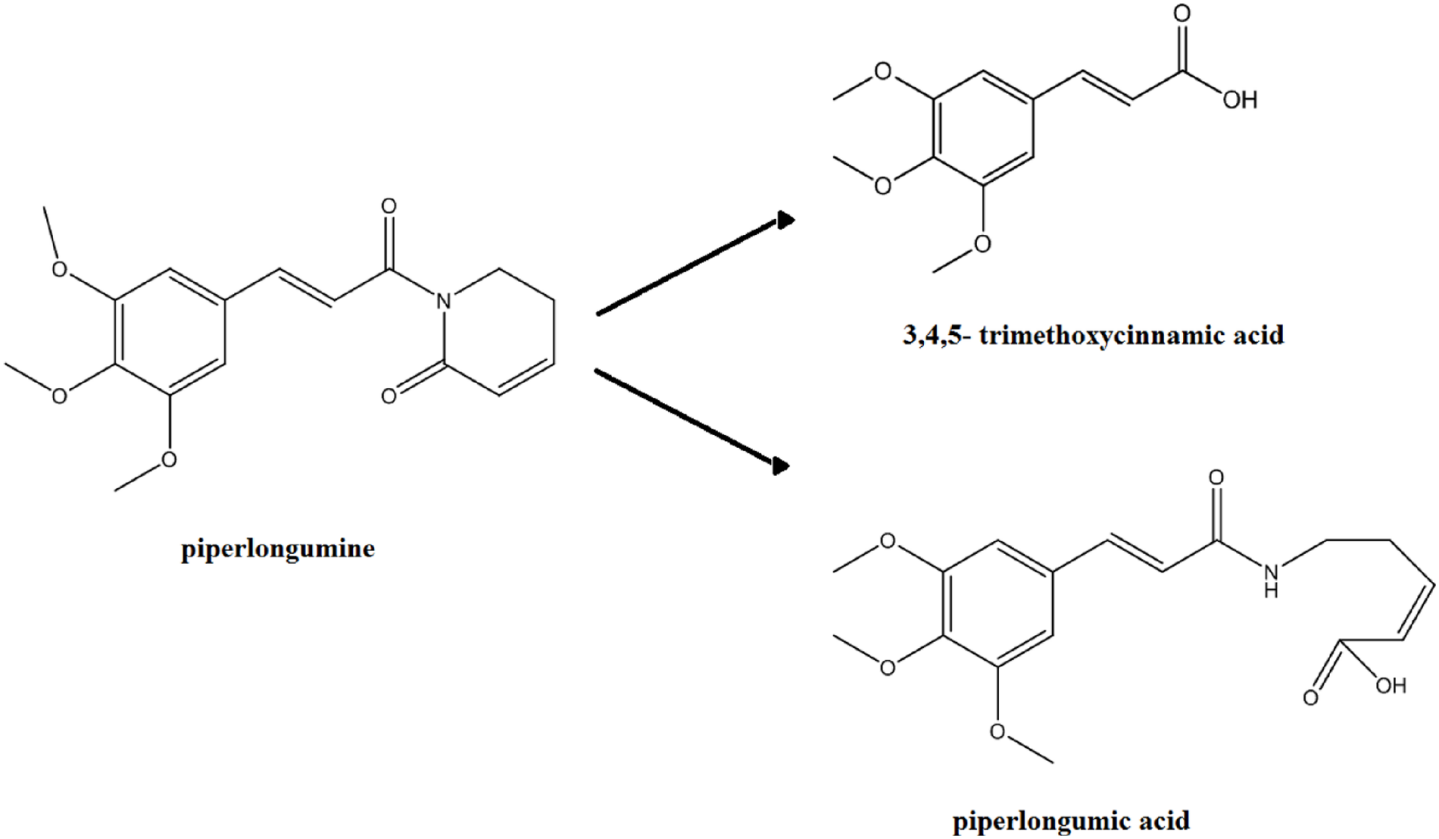
Major degradation products of piperlongumine.

Degradation products were elucidated from stability samples at pH 3, 5 and 8. It was found that 3,4,5- trimethoxycinnamic acid, Fig 12, is a major degradation product. Pure 3,4,5- trimethoxycinnamic acid, externally spiked into stability samples, confirmed that it was the same molecule (retention time approximately 3.1 min). A molecular mass corresponding to what is consistent with piperlongumic acid (see Fig 12) was also isolated (retention time approximately 2.04 min). These findings are in agreement with Chatterjee and Dutta, in which they state that piperlongumine alkaloid undergoes imide hydrolysis in ethanolic alkali to give 3,4,5- trimethoxycinnamic acid as well as piperlongumic acid [19].

### Photo-stability

The results from photo-stability studies (Table 2) indicate that piperlongumine undergoes degradation after exposure to UV light. This was determined by comparing the percentage of drug remaining in the non-light protected samples with their light protected controls. After the exposure duration the concentration of piperlongumine in the control samples remained at 100% of the initial concentration. Interestingly, the greatest degradation occurred in the pure organic solvent, acetonitrile; 60% degradation as compared with approximately 20% occurring in the 15:85 acetonitrile: water samples. Photo-degradation does not appear to be significantly reliant on initial drug concentration, although the samples with the higher initial concentration degraded less than those at the lower initial concentration.

**Table 2:** Summary of photo-stability studies of piperlongumine.

Drug conc. (μg/ml)	% Acetonitrile:water	Average % of drug remaining ± SD
**150**	100:00	44.5 ± 0.2
**150**	15:85	82.3 ± 0.6
**30**	100:00	39.5 ± 0.03
**30**	15:85	79.0 ± 0.1

### Stability with antioxidants

**Fig 13:**
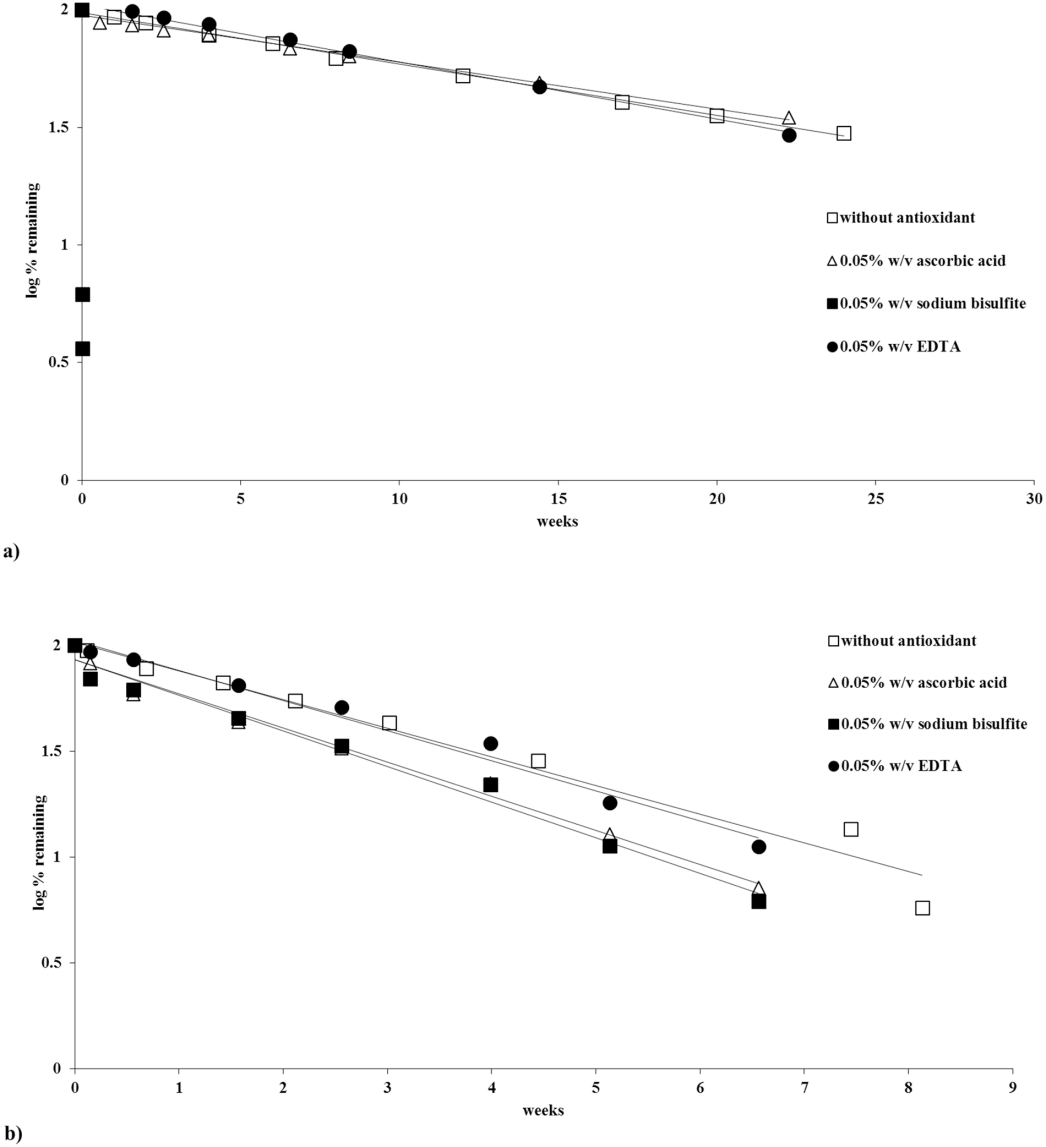
Stability of piperlongumine with the addition of different excipients. (a) pH 7 at 26°C. (b) pH 3 at 56°C.

The addition of ascorbic acid and EDTA to a pH 7 (phosphate buffer) solution did not have a significant effect on the stability profile upon comparing with drug alone at the same condition. This can be seen from the slopes of the graphs in Fig 13a and k values in Table 3. However, the addition of sodium bisulfite degrades piperlongumine very rapidly, within a few minutes, at pH 7. Although sodium bisulfite is routinely used as an antioxidant in pharmaceutical formulations, is has been shown on occasion to decrease the stability of some drugs [20]. Interestingly, the addition of sodium bisulfite with the drug in a pH 3 citrate buffer, at 56°C, did not affect the degradation rate of the drug upon comparing it with drug alone under the same condition (see Fig 13b and Table 3). This finding could indicate that partial ionization of the sodium bisulfite molecule, as would occur at pH 7, plays a part in the rapid degradation of piperlongumine. This pH effect is similar to what has been reported by Scheiner et al. about the stability of thiamine with sodium bisulfite, in which thiamine shows better stability with sodium bisulfite at lower pH (pH 5), but destruction at higher pH (pH 7) [21]. Finally, the addition of ascorbic acid at pH 3 also did not readily affect the degradation of piperlongumine.

**Table 3:** Stability comparisons for piperlongumine with different excipients.

Buffer type	pH	°C	Additional substance to the drug	k values (weeks^-1^)	T_90_ weeks
**Citrate**	3	56	None	0.3107	0.3
**Citrate**	3	56	Ascorbic acid	0.3706	0.3
**Citrate**	3	56	Sodium bisulfite	0.3860	0.3
**Citrate**	3	56	EDTA	0.3266	0.3
**Citrate**	3	26	Hydrogen peroxide	0.3369	0.3
**Phosphate**	7	26	None	0.0494	2.1
**Phosphate**	7	26	Ascorbic acid	0.0459	2.3
**Phosphate**	7	26	Sodium bisulfite	3069.8950	0.0
**Phosphate**	7	26	EDTA	0.0559	1.9

The addition of an oxidizing agent like hydrogen peroxide does significantly increase degradation. The T_90_ with hydrogen peroxide at 26°C is 0.34 weeks. (Table 3) whereas the T_90_ without hydrogen peroxide would be about 9.5 weeks (Table 1).

## Conclusion

Preformulation studies on piperlongumine have been performed. Although it has poor water solubility (0.026 mg/ml), solubility can be increased to 1 mg/ml by using a 20% (w/v) hydroxypropyl-β-cyclodextrin solution or a 10% ethanol: 40% PEG 400 (w/v) formulation. Piperlongumine is more stable in acidic conditions. The T_90_ at pH 4 is about 17 weeks at 25°C, however, it is only a few days at pH 7 and above. The major hydrolysis degradation products of piperlongumine are 3,4,5- trimethoxycinnamic acid and piperlongumic acid. Moreover, it was noted that piperlongumine does undergo photo-degradation. These preformulation studies on the fundamental physicochemical characterizations of piperlongumine can be utilized to assist in the further drug development of this promising therapeutic agent.

## References

[1] Kumar S, Kamboj J, Suman, Sharma S. Overview for Various Aspects of the Health Benefits of Piper Longum Linn. Fruit. J Acupunct Meridian Stud. 2011; 4 (2):134–140. 10.1016/S2005-2901(11)60020-4 21704957

[2] Mishra SS, Tewari JP. Phytochemical Investigation of Piper chaba. J Pharm Sci. 1964; 53, (11): 1423–1424. 14253615 10.1002/jps.2600531138

[3] Adams DJ, Dai M, Pellegrino G, Wagner BK, Stern AM, Shamji AF, et al Synthesis, cellular evaluation, and mechanism of action of piperlongumine analogs. Proceeding of the National Academy of Sciences of the United States of America. 2012; 109 (38): 15115–15120. 10.1073/pnas.1212802109 PMC3458345 22949699

[4] Lee SW, Mandinova A. Patent application title: Methods for the treatment of cancer using piperlongumine and piperlongumine analogs 2009. WO 20090312373.

[5] Roh J-L, Kim EH, Park JY, Kim JW, Kwon M, Lee B-H. Piperlongumine selectively kills cancer cells and increases cisplatin antitumor activity in head and neck cancer. Oncotarget. 2014; 5 (19): 9227–9238. 25193861 10.18632/oncotarget.2402 PMC4253430

[6] Dhillon H, Chikara S, Reindl KM. Piperlongumine induces pancreatic cancer cell death by enhancing reactive oxygen species and DNA damage. Toxicol Reports. 2014; 1: 309–318. 10.1016/j.toxrep.2014.05.011 PMC4268771 25530945

[7] Fofaria NM, Kim S-H, Srivastava SK. Piperine causes G1 phase cell cycle arrest and apoptosis in melanoma cells through checkpoint kinase-1 activation. PLOS One. 2014; 9 (5): e94298 10.1371/journal.pone.0094298 24804719 PMC4013113

[8] Niu M, Xu X, Shen Y, Yao Y, Qiao J, Zhu F, et al Piperlongumine is a novel nuclear export inhibitor with potent anticancer activity. Chemico-Biological Interactions. 2015; 237: 66–72. 10.1016/j.cbi.2015.05.016 26026911

[9] Fofaria NM, Srivastava SK. Critical role of STAT3 in melanoma metastasis through anoikis resistance. Oncotarget. 2014; 5 (16): 7051–7064. 25216522 10.18632/oncotarget.2251 PMC4196183

[10] Fofaria NM, Srivastava SK. STAT3 induces anoikis resistance, promotes cell invasion and metastatic potential in pancreatic cancer cells. Carcinogenesis. 2015; 36 (1): 142–150. 10.1093/carcin/bgu233 25411359 PMC4291051

[11] Raj L, Ide T, Gurkar AU, Foley M, Schenone M, Li X, et al Selective killing of cancer cells by a small molecule targeting the stress response to ROS. Nature. 2011; 475: 231–234. 10.1038/nature10167 21753854 PMC3316487

[12] Patel K, Chowdhury N, Doddapaneni R, Boakye CH, Godugu C, Singh M. Piperlongumine for enhancing oral bioavailability and cytotoxicity of docetaxel in triple-negative breast cancer. J Pharm Sci. 2015; 104: 4417–4426. 10.1002/jps.24637 26372815 PMC4706797

[13] Li J, Sharkey CC, King MR. Piperlongumine and immune cytokine TRAIL synergize to promote tumor death. Nature Sci Rep 2015; 5, 9987; 10.1038/srep09987 PMC4649998 25984950

[14] Son DJ, Kim SY, Han SS, Kim CW, Kumar S, Park BS, et al Piperlongumine inhibits atherosclerotic plaque formation and vascular smooth muscle cell proliferation by suppressing PDGF receptor signaling. Biochemical and Biophysical Research Communications. 2012; 427: 349–354. 10.1016/j.bbrc.2012.09.061 22995306 PMC3495231

[15] Naika R, Prasanna KP, Sujan Ganapathy PS. Antibacterial activity of piperlongumine an alkaloid isolated from methanolic root extract of Piper Longum L. Pharmacophore. 2010; 1 (2): 141–148.

[16] Bezerra DP, Pessoa C, de Moraes MO, Saker-Neto N, Silveira ER, Costa-Lotufa LV. Overview of the therapeutic potential of piplartine (piperlongumine). Eur J Pharm Sci. 2013; 48: 453–463. 10.1016/j.ejps.2012.12.003 23238172

[17] Fofaria NM, Qhattal HSS, Liu X, Srivastava SK. Nanoemulsion formulations for anti-cancer agent piplartine- Characterization, toxicological, pharmacokinetics and efficacy studies. Intl J Pharm. 2016; 498: 12–22. 10.1016/j.ijpharm.2015.11.045 PMC4718800 26642946

[18] Banerjee T, Chaudhuri S. The crystal and molecular structure of N-(3,4,5-trimethoxycinnamoyl) -Δ3- piperidine- 2- one, an amide alkaloid (piperlongumine), C17 H19 NO5. Can. J. Chem. 1986; 64: 876–880.

[19] Chatterjee A, Dutta CP. Alkaloids of Piper Longum linn-I structure and synthesis of piperlongumine and piperlonguminine. Tetrahedron, pergamon press. 1967; 23: 1769–1781. 10.1016/s0040-4020(01)82575-8 6047519

[20] Amidon GL, Connors KA, Kennon L. Chemical stability of pharmaceuticals A handbook for pharmacist. 1st ed John Wiley & sons; 1979 ISBN 0-471-02653-0. P 97.

[21] Scheiner JM, Araujo MM, DeRitter E. Thiamine destruction by sodium bisulfite in infusion solutions. Am. J. Hosp. Pharm. 1981 12; 38(12):1911–3. 7325171

